# INTERGENERATIONAL TUTORING: HEALTH BENEFITS FOR OLDER ADULTS IN A TWO-YEAR QUASI-EXPERIMENTAL STUDY

**DOI:** 10.1093/geroni/igae098.3861

**Published:** 2024-12-31

**Authors:** Peter Sun, Nancy Morrow-Howell, Mary Click, Kendra Minch

**Affiliations:** Bar-Ilan University, Ramat Gan, Tel Aviv, Israel; Washington University in St. Louis, St. Louis, Missouri, United States; The Oasis Institute, St. Louis, Missouri, United States; The Oasis Institute, St. Louis, Missouri, United States

## Abstract

The COVID-19 pandemic has significantly impacted older adult volunteering. It is important to understand if prior relationships between volunteering and health remain consistent in a post-pandemic context. We surveyed Oasis tutors aged 51 and over before and after two years of volunteering and used three matching algorithms—nearest-neighbor matching, optimal full matching, and subclassification—to match them with a comparison group of non-volunteers in the 2016 and 2018 waves of the Health and Retirement Study. G-computation was then used on the merged sample (N = 518) to estimate the effect of volunteering on depression, functional limitations, and self-rated health. With all three matching algorithms, Oasis tutors experienced lower levels of depression and fewer functional limitations, compared to the HRS group of non-volunteers. When using nearest-neighbor matching, Oasis tutors had better self-rated health than the HRS comparison group. Volunteering in the Oasis Intergenerational Tutoring Program positively impacts older adults’ health, supporting the social model of health promotion.

